# Neuroprotection in a Novel Mouse Model of Multiple Sclerosis

**DOI:** 10.1371/journal.pone.0079188

**Published:** 2013-11-04

**Authors:** Katie Lidster, Samuel J. Jackson, Zubair Ahmed, Peter Munro, Pete Coffey, Gavin Giovannoni, Mark D. Baker, David Baker

**Affiliations:** 1 Blizard Institute, Barts and The London School of Medicine and Dentistry, Queen Mary University of London, London, United Kingdom; 2 School of Clinical and Experimental Medicine, College of Medical and Dental Sciences, University of Birmingham, Birmingham, United Kingdom; 3 Institute of Ophthalmology, University College London, London, United Kingdom; Institute Biomedical Research August Pi Sunyer (IDIBAPS) - Hospital Clinic of Barcelona, Spain

## Abstract

Multiple sclerosis is an immune-mediated, demyelinating and neurodegenerative disease that currently lacks any neuroprotective treatments. Innovative neuroprotective trial designs are required to hasten the translational process of drug development. An ideal target to monitor the efficacy of strategies aimed at treating multiple sclerosis is the visual system, which is the most accessible part of the human central nervous system. A novel C57BL/6 mouse line was generated that expressed transgenes for a myelin oligodendrocyte glycoprotein-specific T cell receptor and a retinal ganglion cell restricted-*Thy1* promoter-controlled cyan fluorescent protein. This model develops spontaneous or induced optic neuritis, in the absence of paralytic disease normally associated with most rodent autoimmune models of multiple sclerosis. Demyelination and neurodegeneration could be monitored longitudinally in the living animal using electrophysiology, visual sensitivity, confocal scanning laser ophthalmoscopy and optical coherence tomography all of which are relevant to human trials. This model offers many advantages, from a 3Rs, economic and scientific perspective, over classical experimental autoimmune encephalomyelitis models that are associated with substantial suffering of animals. Optic neuritis in this model led to inflammatory damage of axons in the optic nerve and subsequent loss of retinal ganglion cells in the retina. This was inhibited by the systemic administration of a sodium channel blocker (oxcarbazepine) or intraocular treatment with siRNA targeting caspase-2. These novel approaches have relevance to the future treatment of neurodegeneration of MS, which has so far evaded treatment.

## Introduction

Multiple sclerosis (MS) is an autoimmune, demyelinating disease of the CNS, which is the major cause of non-traumatic disability in young adults. MS is associated with immune-mediated, relapsing-remitting disease [1], followed by or replaced at onset by the development of progressive MS and worsening of disability with or without superimposed relapses [1]. Despite some success at treating relapsing MS with immunosuppressive agents(Coles et al., 2008; Giovannoni et al., 2010; Kappos et al., 2010), control of progressive disability with neuroprotective therapies has yet to be achieved and remains a significant unmet clinical need [1]. Progressive disability is associated with the development of axonal and neuronal loss mediated by immune-dependent and independent mechanisms of neurodegeneration [2,3]. Whilst the failure to control progression may be limited by the identification of suitable modulatory agents, the most appropriate trial to test therapeutic strategies has not been defined [4]. Trials could take many years to complete because of the significant pre-trial recruitment issues owing to the large size of studies required, the long trial duration and the substantial post-trial analysis. Therefore, alternative methods to detect CNS damage and repair need to be developed and evaluated.

The visual system is an accessible sensory system incorporating white matter targeted by neuroinflammatory disease and provides an ideal target to monitor nerve loss and repair. Optic neuritis (ON) occurs as a result of inflammation of the optic nerve and is often the presenting feature of MS [1]. This can result in loss of vision due to impulse conduction failure through demyelination, axonal transection and loss in the optic nerve followed by depletion of retinal ganglion cells (RGC) in the retina [5]. Whilst ON is associated with retinal nerve fibre layer (RNFL) thinning during the progression of MS, people with MS irrespective of a diagnosis of ON develop retinal nerve loss [5]. Therefore, monitoring axonal loss due to ON is a useful way to assess therapeutic strategies targeting neurodegeneration during inflammatory CNS disease.

Experimental autoimmune encephalomyelitis (EAE) is the most intensively used animal model for the study of autoimmune CNS disease. EAE has contributed significantly to our understanding of CNS biology, notably autoimmunity, and has led to the development of several licensed therapies for the treatment of relapsing MS [6,7]. Disease in EAE is associated with a T cell-mediated, ascending paralysis of the tail and hindlimbs [8] and is considered a substantial/severe procedure, owing to the nature of induction with Freund’s adjuvant and the clinical course resulting in suffering to the animal. It is clear that immunosuppression can inhibit relapsing autoimmunity [3,7], however, EAE can also exhibit immune-dependent and autoimmune-independent progression associated with neurodegeneration that may be relevant to progressive MS [3,8,9]. EAE typically affects the spinal cord and although the brain and optic nerves can be involved, optic neuritis is not always a consistent feature [8,10]. However, when present, labelling of RGC by retrograde uptake following surgical brain injection of tracers, it is possible to detect RGC loss, secondary to axonal damage [11].

We therefore aimed to create a novel mouse model of ON to test neuroprotective strategies targeting MS and other inflammatory optic neuropathies. An important additional aim was to develop an alternative model to EAE and therefore refine and reduce the number of animals used in research (3Rs - refinement, reduction and replacement) over conventional models that could be used to serially monitor immune-mediated neurodegeneration, using outcome measures relevant to human disease. 

## Materials & Methods

### Animals

C57BL/6-Tg(Tcra2D2,Tcrb2D2)1Kuch(MOG^TCR^) mice [12] andC57BL/6.Cg-Tg(Thy1-CFP)23Jrs/J*Thy1*CFP mice [13] were obtained from Jackson Laboratories (Bar Habor, Maine USA). These were crossed to create a transgenic MOG^TCR^x*Thy1*CFP line (C57BL/6-Tg(Tcra2D2,Tcrb2D2)1Kuch.Cg-Tg(Thy1-CFP)23Jrs/J) mouse. Animals were maintained in enriched cages on a 12h:12h light:dark cycle with food and water *ad libitum*, as reported previously to conform with the principles of ARRIVE guidlelines [8]. It is known that environment can influence spontaneous disease in T cell receptor transgenic mice [14] and the mice used in this study were housed in pathogen free individually ventilated cages and both male and female animals from 8 weeks old were used in experiments. In accordance with the Animals Act 1986, the procedures were approved by a local, independent ethical committee (Queen Mary University London Ethical Review Panel) and by the UK Government Home Office Inspector. All procedures and housing of animals were performed and controlled under license of the researcher, the project and the Institution issued by the UK Government. The housing/conditions cage sizes and operating procedures are detailed in the reference provided [8], we suggest replication of the model should be achieved in similar conditions.

### Animal phenotyping and genotyping

A quantitative PCR (qPCR) method was developed to identify homozygous mice for breeding. The CFP oligonucleotide sequence was identified from the NCBI Nucleotide library and the MOG^TCR^ transgene was sequenced from PCR products. A duplex qPCR was performed with Ribosomal protein S29 (RPS29) as an endogenous control, which is expressed at a constant level [15]. Oligonucleotide primers and probes based upon enhanced CFP (eCFP) (eCFP forward primer, GCCTACATACCTCGCTCTGC; eCFP reverse primer, CAACCCGGTAAGACACGACT; eCFP probe [FAM]-ATCCTGTTACCAGTGGCTGC-[TAM]), MOG^TCR^ (MOG^TCR^ forward primer, ACCCAGTGGTTCAAGGAGTG; MOG^TCR^ reverse primer, CTTGGTTCCCTGTCCAAAGA; MOG^TCR^ probe [FAM]-AGCGACTGGGCTGTGTACTT-[TAM]) and RPS29 (RPS29 forward primer, ACGGTCTGATCCGCAAATAC; RPS29 reverse primer CATTCAAGGTCGCTTAGTCCA; RPS29 probe [HEX]-TACGCGAAGGACATAGGCTT-[TAM]) were used in qPCR. The target gene probes were made with the fluorescent dye FAM attached to the 5’ end and a fluorescent quencher TAMRA attached to the 3’ end. The reference gene probes were made with HEX attached to the 5’ end to allow distinction between target and reference gene. Data was collected on 7500 System Sequence Detection Software (Applied Biosystems, Warrington, Cheshire, UK) and cycle threshold (C_t_) values were analysed by using a manual baseline of 0.2. Results were exported and analysed using CopyCaller™ software to determine number of transgenes. 

### Induction of ON

Animals were injected intraperitoneally (i.p.) on day 0 and 2 with 150ng *Bordetella pertussis* toxin (PTX Sigma, Poole, UK) and on day 14 with 0.25mg MOG-specific Z12 mouse IgG2a monoclonal antibody, which was protein-G (GE Healthcare Life Sciences, Buckinghamshire, UK) purified from tissue culture supernatant or supplied by Dr. P. Smith, UK [16]. *Bordetella pertussis* toxin is required to induce consistent disease, as show previously in single MOG^TCR^ transgenic mice [17]. Animals were monitored daily to assess the development of paralysis, typical of EAE. These were scored 0= normal, 1 = limp tail, 2 = impaired righting reflex, 3= hindlimb paresis, 4 = complete hindlimb paralysis, 5= moribund [8]. Animals were killed either by cervical cord dislocation of C0_2_ overdose. 

### Anaesthesia

Animals were anaesthetised with an i.p. injection of 75mg/kg ketamine and 1mg/kg medetomidine in normal saline and reversal was achieved with 1mg/kg atipamezole. Eyes were dilated with one drop of tropicamide (Minims® tropicamide 1% w/v) and one drop of phenylephrine hydrochloride (Minims® phenylephrine hydrochloride 2.5% w/v). A drop of 2% hydroxypropyl methylcellulose was used to prevent cataract formation during anaesthesia.

### Measurement of visual sensitivity

Visual sensitivity was measured using a visual-tracking drum, which consisted of a motorised drum with black and white vertical stripes, which rotated in a clockwise and anti-clockwise direction at a frequency of 0.5 cycles/degree [18]. The mouse was placed on a stationary platform in the centre of the drum and was not restrained allowing freedom of movement. An initial settling period of 30 seconds was followed by a pattern of 60 second clockwise rotations, a 30 second intermediary pause and 60 second anti-clock wise rotations, repeated twice. Successful ‘head tracking’ movement was characterised as a horizontal head movement in the direction of rotation at the same speed as the visual tracking drum. Analysis of head tracking movements was performed blinded by analysis of video recordings.

### Measurement of visual evoked potential (VEP)

Anaesthetised animals were secured in a stereotaxic frame and body temperature was maintained at 35°C with a small heating plate with built-in resistance temperature detector sensor connected to a direct current temperature controller. Animals were dark adapted for 30 minutes and remained in the dark between flashes. The active electrode was a needle electrode placed subcutaneously (s.c.) over the visual cortex and the reference electrode was a needle electrode placed s.c. in the snout. The signal was amplified 10,000x (NL104A AC Amplifier, Digitimer, UK) and a flash stimulus of 10ms duration every 300ms from a stand-alone monocular ganzfield photic stimulator (MGS-2, LKC Technologies, MD, USA) was presented approximately 20cm from eyes at a flash intensity of -10dB (0.2436cd.sec/m^2^). Signals were band pass filtered at 5Hz and 1kHz and sampled at 1000 Hz. Recordings were made with Signal v3.11 and for each recording 200 stimuli were captured and consecutive sweeps averaged to produce a mean VEP response. Latency was measured between N1 and P2 (ms) and amplitude was measured between N1 and P2 (μV).

### Measurement of RGC loss using scanning laser ophthalmoscope (SLO) and RNFL thinning using optical coherence tomography (OCT)

A Multiline OCT was modified from a Spectralis® HRA + OCT (Heidelberg Engineering, Heidelberg, Germany) to comply with mouse optics. Modifications included a reduction in the diameter of the excitation laser beam to 2mm to allow more efficient coupling of laser light into the small aperture of the mouse eye and addition of a +25 diopter add-on lens to correct for the characteristic hyperopia in mice (Heidelberg Engineering). In addition, the standard chin rest was replaced with a special custom-built (Institute of Ophthalmology workshop, London, UK) animal mount to allow the animal to be positioned correctly. To allow visualisation of CFP-expressing RGC (460nm excitation and 490nm detection) a RazorEdge® ultrasteep long-pass filter, which transmitted between 458 and 670nm (LP02-458RS-25, Semrock, Baltimore, USA) was inserted into a filter slot to allow detection of CFP.

Examinations were recorded in both right (oculus *dextrus*, OD) and left eyes (oculus sinister, OS) of each animal at day 0 and day 21 after immunisation. To capture an OCT image, animals were anaesthetised and placed on the animal mount and an infra-red (IR) reflection image with the optic nerve head in a centralised position was achieved with optimal focus (approx +18.0 dioptres). A RNFL single exam using the automatic real time (ART) mode (allows averaging of 100 recordings) was produced for each mouse eye, which automatically measured RNFL thickness (µm) in a 30° circle surrounding the optic nerve head. To capture an SLO image, an IR reflection image was achieved as described previously and the CFP (450nm) laser was selected to allow CFP-expressing RGC to be visualised. A constant sensitivity of 80%, a field view of 30° and a reference arm parameter of 28616µm [28.616mm] were maintained for each eye examined. To calculate the RGC density, images were analysed in a blinded fashion using Image J software. Assumptions on the area of retina observed were made from [19], who estimated 1° of field is subtended by 30µm of retina, therefore, a 30° field of view is subtended by 900µm area of retina. The numbers of RGC were counted in the superior, nasal, inferior and temporal quadrants and the total density of RGC calculated per mm^2^. It has been shown that 95% of retinal CFP positive cells are RCG [20]. Whilst amacrine cells are labelled with CFP, these are found in a different layer of the retina and are therefore not visualized when viewing RGC.

### Histological assessment

Animals were transcardially perfused with phosphate buffered saline (PBS) and Karnovsky’s fixative. Tissue was post-fixed using 1% aqueous (w/v) osmium tetroxide and was resin embedded. Semi-thin sections were cut at 0.7µm thickness and ultra-thin sections were cut between 80-90nm with a 6mm Ultradiamond knife. Semi-thin sections were stained with 1% toluidine blue and ultra-thin sections were stained with Reynold’s Lead Citrate [10]. Samples were viewed on a Nikon Eclipse 80i microscope and JEOL1010 transmission electron microscope.

### Preparation of retinal flatmounts

Eyes were enucleated and immersed in 4% PFA (Sigma, Poole, Dorset, UK) in PBS (pH7.4) overnight. The retinae were dissected and the cornea, sclera, lens, hyaloid vasculature and connective tissue were removed. Four radial incisions were cut around the retinae and flatmounts were mounted onto slides and cover slipped with anti-fade glycerol (CitiFluor Ltd, London, UK). The retinal flatmounts were imaged by fluorescent microscopy and RGC density was calculated by counting CFP expressing RGC using Stereo Investigator 7.35.1. RGC were counted by drawing a contour at 10x magnification around the retinal nerve flatmount. A grid was created and a fractionator probe was used to create 10 random counting frames (100µm x 100µm). RGCs were counted at 40x magnification in each randomly selected 100µm^2^ box. The total numbers of RGC was calculated from the sample of 10 squares and an approximation of the density in the retinal flatmount was calculated. The quanitification was carried out blinded to the observer.

### Immunohistochemistry

Frozen sections were thawed for 30 minutes at room temperature and then immunostained for active caspase-2 using a 1:200 dilution rabbit polyclonal IgG (ab2251 - Abcam, Cambridge, UK) and total caspase-2 using a 1:400 dilution rabbit polyclonal IgG (sc-623 Santa Cruz), with a secondary antibody Alexa Fluor 568 donkey anti-rabbit IgG (H+L) at a 1:1000 dilution (A-21206. Invitrogen, Paisley, UK). Sections were imaged using a Zeiss LSM 510 confocal laser scanning microscope [21]. 

### Neuroprotective study of oxcarbazepine

Oxcarbazepine (OXC) (Sigma-Aldrich Ltd, Poole, Dorset, UK) was administered in a 1:1 dilution of PBS and DMSO (dimethyl sulfoxide) and administered i.p. daily in 100µl at a dose of 10mg/kg. This drug dose was chosen based on those used in other neuroprotection studies. Animals treated with vehicle were injected i.p. daily with 100µl of 1:1 dilution of PBS and DMSO. Analysis was performed blinded to treatment. Animals were randomized to either treatment or drug within a cage. The primary outcome was number of RGC in retinal flatmounts as this was undertaken before acquisition of the OCT/SLO device.

### Intravitreal injection caspase-2 siRNA

Small interfering RNA (siRNA) targeting caspase-2 and control nonsense siRNA were provided by Quark Pharmaceuticals, California, USA as described previously [20]. A fine tip glass pipette was used to inject a total of 2μg of caspase-2 siRNA dissolved in 2μl of physiological saline into the mouse eye. The mouse was briefly anaesthetised in an isofluorane chamber until eye flick response had stopped and the mouse showed signs of slowed breathing. Animals were intravitreally injected through the sclera immediately posterior to the limbus in both eyes on day 0 and 10 after ON disease (siRNA) induction. Analysis was performed blinded to treatment.

###  Statistical Analysis

Statistical analysis was performed using SigmaStat 3.1. Results were presented as mean values ± SEM. Differences between outcome measures (visual sensitivity, VEP amplitude and latency, OCT and SLO) for day 0 (before ON) and day 21 (after ON) were analysed using a paired t-test. Correlations were analysed using the Pearson product moment correlation. Results was considered significantly different if the probability level *P*<0.05 (*), *P*<0.01(**) or *P*<0.001(***) was reached between groups.

## Results

### Characterisation of MOG^TCR^xThy1CFP mouse model

**Figure 1:**
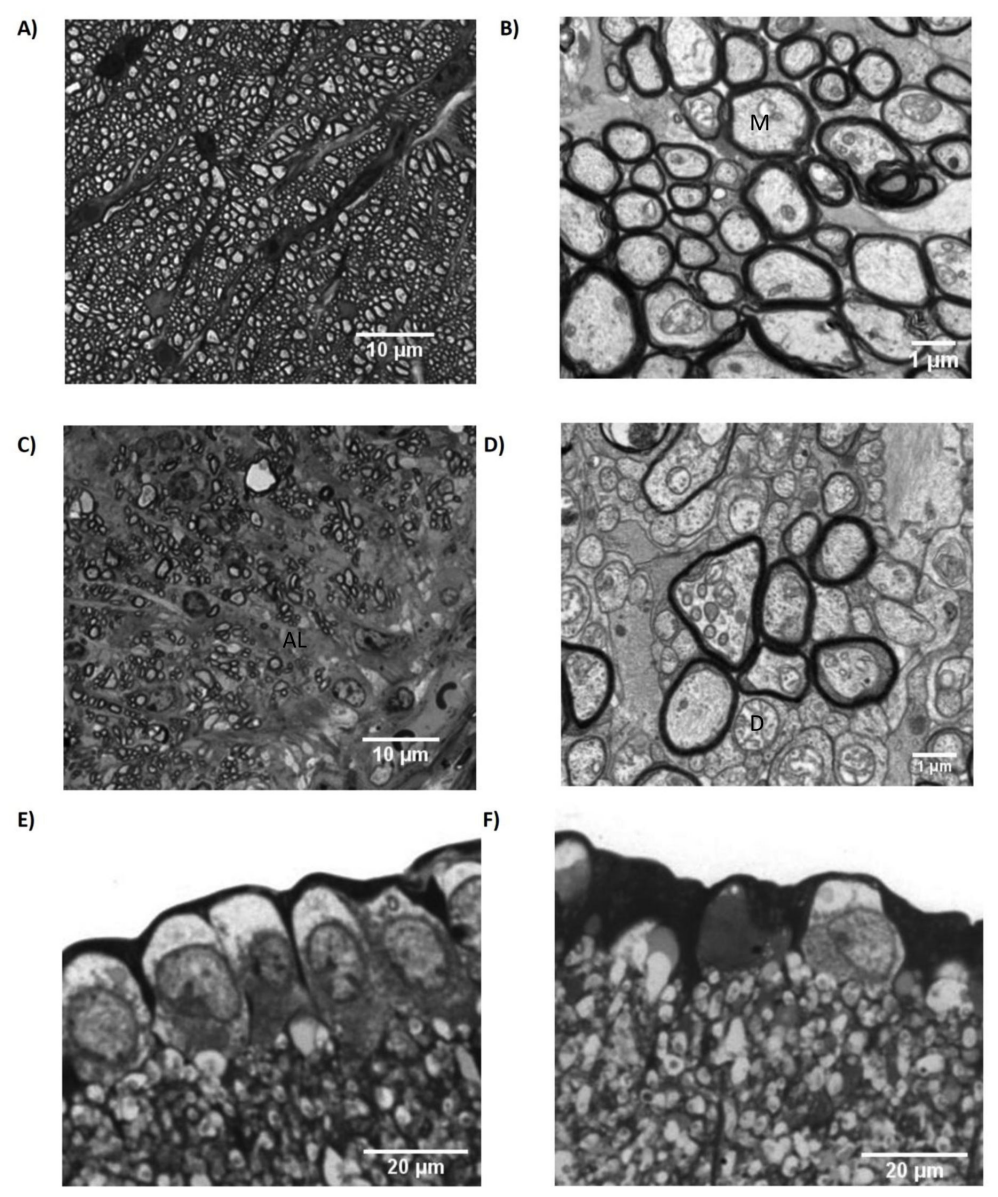
Optic and retinal nerve loss during ON in MOG^TCR^xThy1CFP mice. (A) Semi-thin toluidine blue stained optic nerve cross section and (B) ultra-thin lead citrate stained sections from wildtype mice showing uniformly myelinated axons (M). (C) Semi-thin toluidine blue stained section and (D) ultra-thin lead citrate stained section of optic nerves from immunized MOG^TCR^xThy1CFP developing ON on day 21, showing evidence of demyelination (D) and areas of axonal loss (AL). (E) Semi-thin toluidine blue stained cross section of retina from wildtype mouse, showing healthy RGC (RGC) and (F) semi-thin toluidine blue stained cross section of retina from immunised MOG^TCR^xThy1CFP mice developing ON on day 21 showing irregular RGC.

**Figure 2:**
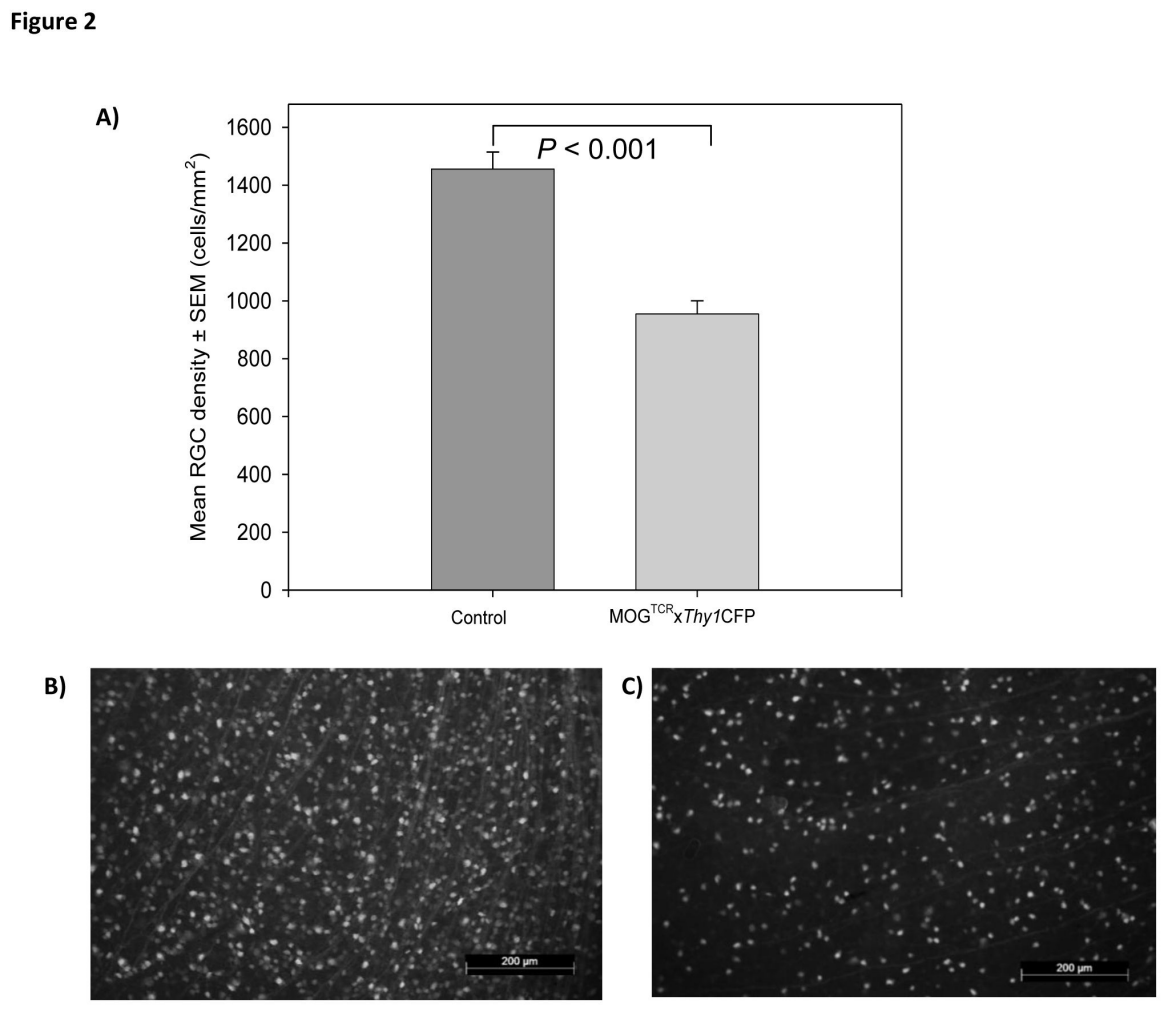
RGC loss during ON in MOG^TCR^xThy1CFP mice. (A) Decrease in mean RGC density after immunisation of MOG^TCR^xThy1CFP mice (n=7) compared to control mice (n=7) at day 21. (B) Retinal flatmount of control mouse at 20x magnification showing a high density of CFP expressing RGC and (C) retinal flatmount of retina after immunisation of MOG^TCR^xThy1CFP mice showing a low density of CFP expressing RGC.

H2-A^b^-restricted, myelin oligodendrocyte glycoprotein (MOG)-specific TCR transgenic C57BL/6-Tg(Tcra2D2,Tcrb2D2)1Kuch (MOG^TCR^) mice (which has previously been described and characterised [17]), were crossbred with C57BL/6.Cg-Tg(Thy1-CFP)23Jrs/J (*Thy1*CFP) transgenic mice that expressed neuronally-restricted, *Thy1*(CD90)-promoter driven expression of CFP [13]. This created a double transgenic MOG^TCR^x*Thy1*CFP mouse, which can develop spontaneous, and induced ON and express CFP in the RGC. Approximately 30% of the MOG^TCR^x*Thy1*CFP mice develop spontaneous histological ON with a wide range of time to onset (from 2.5 months to 5 months) consistent with that reported previously for MOG^TCR^ mice [12]. Therefore, an immunising or induction protocol was developed to produce high disease incidence and augment the demyelination potential. Consistent with that reported previously [11,17] spontaneous disease occurred in about 5% of animals and the incidence of optic neuritis was increased following induction with *Bordetella pertussis* toxin [11,17]. Animals were immunised with *Bordetella pertussis* toxin to induce ON and blood:optic nerve barrier dysfunction and then Z12 MOG-specific demyelinating monoclonal antibody (mAb) [16,22]was administered. Although the intention here was to induce ON in the absence of paralytic EAE, it was possible to induce classical paralytic EAE in these animals through administration of additional doses of MOG-specific demyelinating mAb through augmentation of sub-clinical spinal cord lesions (see Table S1) or using active immunisation of MOG-peptide in Freund’s adjuvant [17], should the researcher wish to use this clinical outcome. This mAb treatment also augments classical EAE, often to lethal disease [22]. However, through titration of the amount of MOG-specific mAb, it was possible to induce a milder disease with high incidence without paralytic disease, which carried additional animal welfare benefits. The optic nerve of wild type mice showed normal appearance of large numbers of axons (Figure 1A) that were well myelinated (Figure 1B). However, with the development of ON in MOG^TCR^x*Thy1*CFP mice, fewer numbers of axons were present in affected optic nerves (Figure 1C) with evidence of demyelinated axons (Figure 1D). Although demyelination was present, this was not quantitated in the present study. Using histology, the presence of glial processes within demyelinated, particularly small diameter axons, make the assessment of demyelination very difficult. A 33±18% loss of axons from the optic was associated with a 25±8% loss of retinal ganglion cells assessed in sections of the eye. However, demyelination as assessed by increased latency in electrophysiological readings made some form of quantitation possible and was reported. Further studies are required to improve the amount of demyelination with axonal preservation as loss of myelin is associated with rapid axonal loss, as occurs in classical EAE [6]. A uniform appearance of RGC were present in the ganglion cell layer of the retina in wild type mice (Figure 1E), while ON in mice resulted in significant (*P*<0.005) RGC loss (Figure 1F). This represented about a 35% loss in RGC density (Figure 2A), quantified from retinal flatmounts in healthy controls (Figure 2B) compared to MOG^TCR^x*Thy1*CFP (Figure 2C) mice. It was possible to induce non-paralytic, neuroinflammatory disease in the absence of Freund’s adjuvant and the loss in RGC number could be detected without the need for surgical labelling through injections of tracers into the brain. Having established a method for induction of disease, the application of non-invasive, quantitative outcome measures were investigated, that would contrast the non-parametric and non-linear neurological paralysis scales used in classical EAE [8]. 

### Development of non-invasive methods of disease assessment

#### Visual-tracking drum

**Figure 3:**
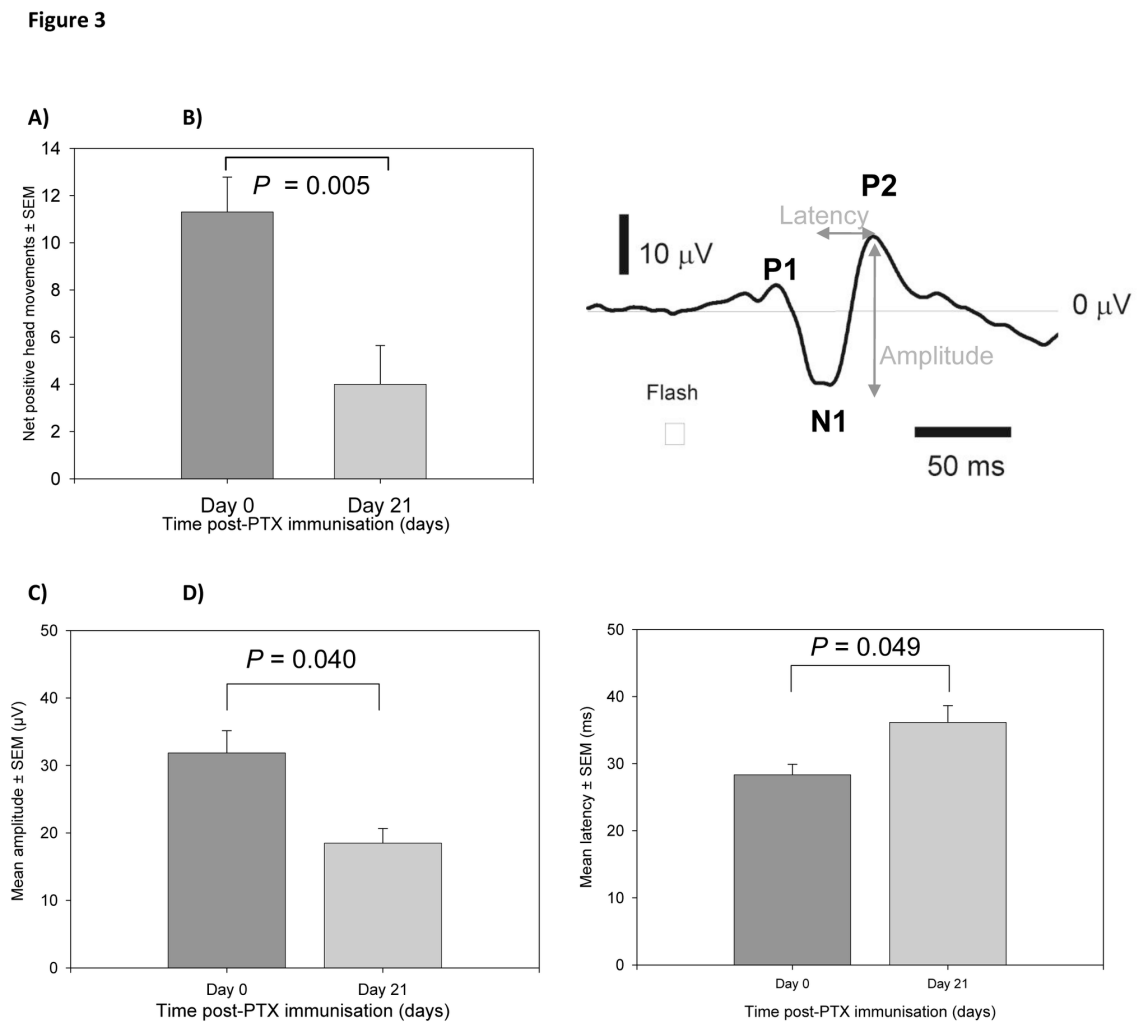
Visual sensitivity loss and electrophysiological changes during ON in MOG^TCR^xThy1CFP mice. (A) Decrease in visual sensitivity after immunisation of MOG^TCR^xThy1CFP to develop ON (n=10). Results represent the mean ± SEM of net positive head movements. **P<0.01 compared to control. (B) Typical waveform VEP showing P1 (first positive peak), N1 (first negative peak), P2 (second positive peak). (C) Decrease in mean VEP amplitude after immunisation of MOG^TCR^xThy1CFP mice to develop ON (n=5). (D) Increase in mean N1-P2 latency of VEP amplitude after immunisation of MOG^TCR^xThy1CFP mice to develop ON (n=5). Results represent the mean ± SEM of amplitude. *P<0.05 compared to control.

A visual-tracking/optokinetic drum can be used to examine visual sensitivity by measuring positive head movements in response to visual stimulation, provided by a vertical bar on the rotating drum. This test assessed bi-lateral vision and was not adjusted to detect the vision in individual eyes [17]. Visual sensitivity was assessed in MOG^TCR^x*Thy1*CFP mice on day 0 (before disease onset) and day 21 (after disease onset), using blinded assessment of video recordings of head movements in relation to the direction of the rotating drum. A significant reduction (*P*<0.01) in the number of positive head movements was observed between day 0 and day 21 (Figure 3A), with a lower incidence of positive head movements occurring on day 21. These results show a decrease in visual sensitivity following the development of ON in MOG^TCR^x*Thy1*CFP mice, indicating functional vision loss.

#### Electrophysiology

Electrophysiology can be used to examine changes in electrophysiological function of the optic nerve by measuring visually evoked potentials (VEP) in response to a flash stimulus. The VEP response was measured in anaesthetised, MOG^TCR^xT*hy1*CFP animals on day 0 (before disease onset) and day 21 (after disease onset) and VEP recorded at a flash intensity of -10dB to assess changes occurring through the development of ON. Recording from the scalp immediately above the occipital lobe, with a reference electrode at the snout, the typical VEP waveform recorded consisted of a small positive peak (P1), a large negative peak (N1) followed by a large positive peak (P2) (Figure 3B). VEP recordings showed a significant decrease (*P*=0.040) in mean amplitude between day 0 and day 21 with a 42% reduction in mean amplitude (Figure 3C). A significant 28% increase (*P*=0.049) in mean latency between day 0 and day 21 was observed (Figure 3D). These results demonstrate that the electrophysiological function of the optic nerve is dysfunctional at day 21 due to disease activity resulting in reduced amplitude and increased latency, correlating with axonal loss and demyelination in the optic nerve, respectively. 

#### Optical coherence tomography

**Figure 4:**
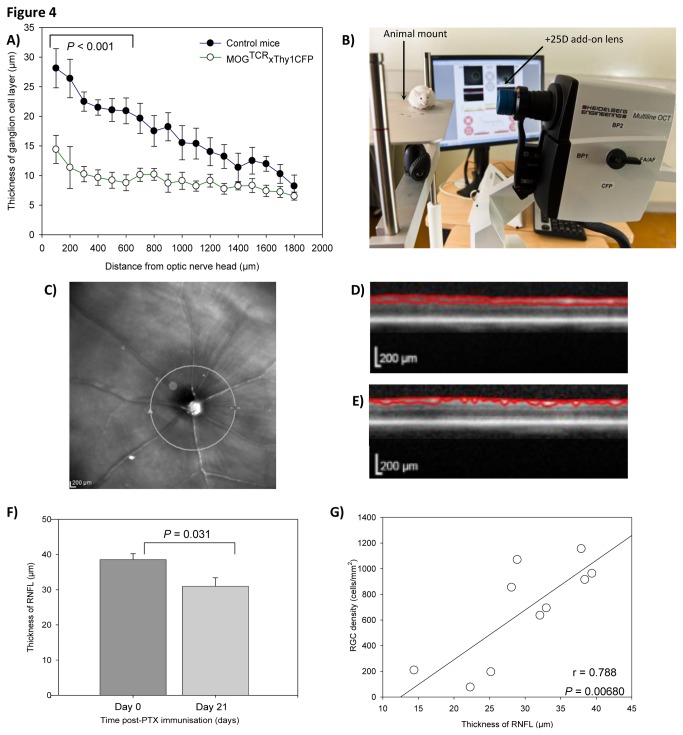
OCT imaging during ON in MOG^TCR^xThy1CFP mice (A) Histological changes in the thickness of RGC layer in control mice (n=5) and MOG^TCR^xThy1CFP mice (n=5) at day 21. Results represent the mean ± SEM of RGC layer thickness. **P<0.01 and ***P<0.001 between control mice and MOG-specific TCR mice at the same distance from optic nerve head. (B) Multiline OCT, a Spectralis® HRA + OCT modified by Heidelberg Engineering Inc to allow imaging of CFP-expressing RGC and adapted to comply with mouse optics with a +25D lens and fitted animal mount. Mouse is ABH strain. (C) OCT images of retina were acquired using a circular OCT scan surrounding the optic nerve head on (D) day 0 and (E) day 21 of disease induction and RNFL thickness was calculated. (F) Decrease in RNFL thickness after immunisation of MOG^TCR^xThy1CFP mice to develop ON (n=10). (G) Correlation between RGC density (measured using retinal flatmounts) and RNFL thickness (measured using OCT). Results represent the mean ± SEM of RNFL thickness. * P<0.05, **P<0.01, ***P<0.001 compared to control.

Optical coherence tomography (OCT) is used in MS and other diseases to detect RGC loss by measuring changes in the thickness of the retinal nerve fibre layer (RNFL) [23,24]. Initial studies using histological examination of retinal sections at the level of the optic nerve head demonstrated that significant loss of RGC occurred and that there was retinal thinning (Figure 4A). A clinical OCT machine with a resolution of less than 3 μm (Spectralis® HRA + OCT, Heidelberg Instruments, Germany), modified to facilitate imaging of rodent eyes was obtained during the course of this study (Figure 4B). Examination by OCT in MOG^TCR^x*Thy1*CFP animals on day 0 (before disease onset) and day 21 (after disease onset) was used to analyse the impact of disease on the reduction of RNFL thickness as a result of RGC loss. The thickness of the RNFL in a defined circle surrounding the optic nerve head was measured (Figure 4C). RNFL thickness at day 0 (Figure 4D) decreased by 12μm after ON in MOG^TCR^x*Thy1*CFP animals (Figure 4E), representing a 32% decrease in RNFL thickness (Figure 4E and F). The thickness of RNFL positively correlated with the density of RGC in retinal flat mounts (Figure 4G) and thus further validates the use of OCT as a surrogate outcome to detect nerve loss in real-time. 

#### Confocal scanning laser ophthalmoscope

**Figure 5:**
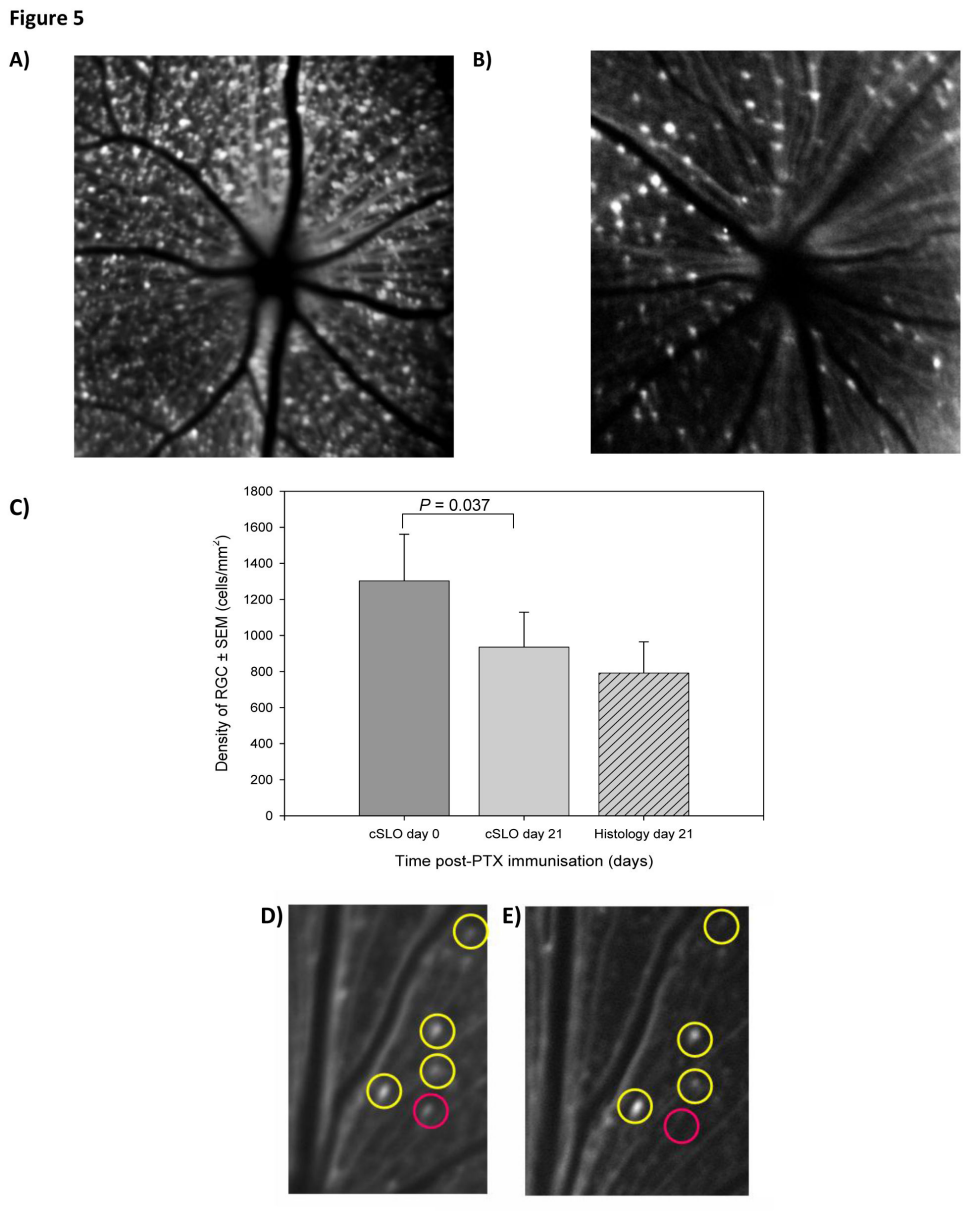
cSLO imaging during ON in MOG^TCR^xThy1CFP mice. (A) Example of retina before disease induction on day 0 and (B) after disease induction on day 21. (C) Quantification of mean RNFL thickness by cSLO on day 0 and after ON disease induction at day 21 and comparison with conventional histology at day 21 (n=8). Images of individual RGC taken over a 30° field of view and a contrast sensitivity of 80% at (D) day 0 and (E) day 21. RGC survival (yellow) and RGC loss (red) are identifiable. Results represent the mean ± SEM of RGC density.

Whilst studies in humans rely on OCT imaging, because of the CFP transgene in MOG^TCR^x*Thy1*CFP mice, it is possible to non-invasively image individual nerves within the eye using a confocal scanning laser ophthalmoscope (cSLO) to produce high-quality magnified images [18]. Animals were anaesthetised to obtain high quality images that were centred around the optic nerve head. The cSLO was used to take images of the retina of MOG^TCR^x*Thy1*CFP on day 0 (Fig. 5A) and day 21 (Fig. 5B), which allowed RGC density to be quantified before and after disease induction (Fig.5C) and compared to conventional histological assessment (retinal flatmounts) of RGC loss. The density of RGC assessed by cSLO was significantly reduced at day 21 compared to day 0 (*P*<0.037). The density of RGC detected by cSLO at day 21 (935±193 cells/mm^2^) correlated well with that determined by histology (792±173 cells/mm^2^) and therefore suggests that the use of cSLO to quantify RGC loss appears to be an accurate method, agreeing with results obtained from histological methods used previously. Using a 15° field of view, the cSLO can also be used to detect individual RGC and areas of RGC loss in the same mouse by using the topography of the blood vessels to locate RGC at day 0 (Figure 5D) and at day 21 (Figure 5E). The results acquired using OCT and cSLO to study the level of RGC loss in MOG^TCR^x*Thy1*CFP mice are concurrent; the decrease in the thickness of the RNFL using OCT was 32±10% and the decrease in RGC density using cSLO was 29±10%. These results provide further evidence that measuring RGC loss using cSLO and RNFL thinning using OCT are accurate methods to assess the disease process.

### Therapeutic control of MOG^TCR^xThy1CFP: use as a neuroprotective model

To validate the MOG^TCR^x*Thy1*CFP model as a useful model to detect and manipulate neuroinflammatory disease two approaches using novel reagents were used. This involved the systemic and local delivery of agents. Whilst there are an increasing number of immunosuppressive agents that can inhibit the relapsing component of MS, none have been shown to control the neurodegenerative aspects of progressive MS [1,25]. 

#### Systemic delivery of a neuroprotective agent

**Figure 6:**
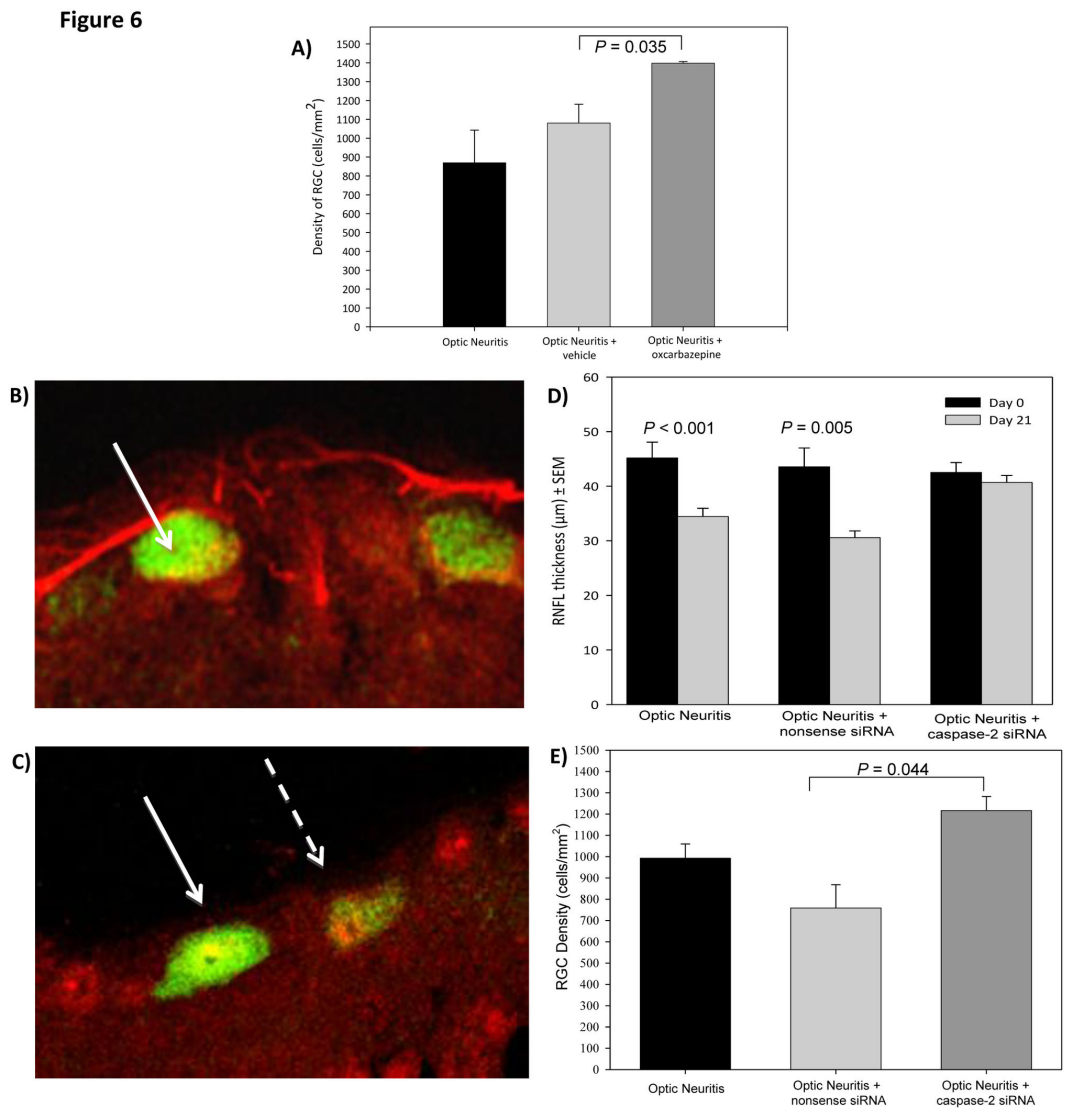
Neuroprotection with oxcarbazepine and caspase-2 siRNA in MOG^TCR^xThy1CFP mice. (A) MOG^TCR^xThy1CFP transgenic mice received the following: optic neuritis control (n=7), 100µl daily dose of vehicle (1:1 PBS and DMSO) (n=7) or 100μl 10mg/kg OXC (dissolved in 1:1 PBS and DMSO) (n=7). Mice were sacrificed on day 21. Eyes were flatmounted and RGC were counted using stereology software. Results represent mean ± SEM of RGC density. (B) Cross sections of the retina stained with total caspase-2 and (C) active caspase-2 and both were secondary labelled with Alex Fluor 568 (Red). RGC are shown in green (arrow), but degenerating RGC with lower CFP expression could be detected (dashed arrow). (D) RNFL thickness after ON induction and treatment with either nonsense or caspase-2 siRNA. (E) Quantification of RGC density after ON induction and treatment with either a nonsense (n=7) or caspase-2 siRNA (n=8). Plots show mean ± SEM of RGC density and RNFL thickness.

It has been suggested that mitochondrial dysfunction due to low grade inflammation can lead to sodium loading and the triggering of death effector pathways leading to nerve death [26,27]. To explore this potential we used the sodium channel blocker Oxcarbazepine (OXC) (10,11-Dihydro-10-oxo-5h-dibenz[b,f]azepine-5-carboxamide). Following the development of neuroinflammatory disease treatment with 10mg/kg/day OXC resulted in significant sparing of RGC compared to treatment with vehicle (Figure 6A). The density of RGC was significantly higher (P<0.05) following OXC treatment, with a RGC density of 1398±9cells/mm^2^, compared to 1080±100cells/mm^2^ in the vehicle group. Therefore, OXC showed the potential to increase the survival of RGC and encouraging neuroprotective potential in the MOG^TCR^x*Thy1*CFP model.

#### Local Delivery of a neuroprotective agent

The eye is a CNS structure that is uniquely accessible to local treatment; we therefore assessed this approach using a novel siRNA. Caspases are death effector molecules that can trigger nerve death in EAE [20]. Caspase-2 was found to be expressed and cleaved (activated) in >95% of RGC after optic nerve crush injury [20]. Immunohistochemistry to detect the expressed caspase-2 (using an antibody against the propeptide of caspase 2) and the cleaved activated caspase-2 (using an antibody against the carboxyterminals aspartate D316 on activated caspase-2) demonstrated that expressed and active caspase-2 was present in the RNFL and in particular, in axons and cell bodies of RGC in MOG^TCR^x*Thy1*CFP mice after ON (Figure 6B and C). This indicated that casapase-2 may play a role in the apoptotic pathway of RGC degeneration in the MOG^TCR^x*Thy1*CFP model of disease. The presence of caspase-2 has previously been shown to be downregulated by intravitreal injection of a chemically modified synthetic siRNA, persists and acts locally within the eye for over 30 days [20]. Analysis of the RNFL thickness using OCT demonstrated a significant inhibition of nerve cell loss following caspase-2 siRNA administration (Figure 6D). Both control and vehicle groups showed a significant decrease in RNFL thickness, 21±6% and 27±7% respectively, after induction of ON as measured at day 0 and day 21. Caspase-2 siRNA treatment inhibited the decrease in RNFL thickness and showed a negligible decrease of 3% compared to pre-disease levels. Treatment with capase-2 siRNA had a significant effect on the increased survival of RGC after ON in MOG^TCR^x*Thy1*CFP mice compared to those treated with control or a vehicle (Figure 6E). This suggests that inhibition of caspase-2 activation using a therapeutic siRNA may be a useful approach that could be useful in the treatment of optic neuritis and other inflammatory optic neuropathies in humans.

## Discussion

Experimental autoimmune encephalomyelitis has been one of the main animal models to investigate autoimmune function [6]. Although many uses of EAE have revolved around inhibiting autoimmunity, the success at treating relapsing-remitting MS, has moved the focus towards monitoring and treating progressive MS. The visual pathway is receiving increased attention as a target tissue to monitor neurodegeneration in MS because of the potential of OCT as a method to detect RNFL thinning [23,24]. We therefore developed a novel ON model of MS, with multiple outcomes such as electrophysiology, visual sensitivity, cSLO and OCT that reflects potential outcome measures for use in human trials. Following pupil dilation the cornea of the eye can show a transient clouding and it is therefore technically challenging to perform electrophysiology and ocular OCT and cSLO imaging at the same time. At the time of these experiments the number of anaesthetics that the animals could undergo was restricted and some of the equipment used for functional testing had not yet been obtained, meaning it was not possible to also obtain functional readouts on the same animals in this study. Use of OCT is a faster procedure than performing quantitative cSLO. This study has highlighted potential different outcomes that can be applied in future studies. These can be selected such that they are appropriate for their target. However, even without additional imaging or other outcomes the model can be used as a screening tool or having a 3Rs-refined outcome to detect the influence of transgenesis. We have demonstrated two therapeutic strategies that can protect against nerve cell loss in this model. Both these therapeutic strategies could be developed clinically. 

This current study describes a refined model for immunological and neuroprotection studies, further work may refine the model more. The model is currently sufficiently robust to detect the action of therapeutic agents such that the approach may be directly translated to MS, as optic neuritis is increasingly been seen as a major target. The disease is consistently robust and exhibits a high incidence and consistent severity, which is not always the case in MOG peptide and Freund’s adjuvant induced disease [28,29]. Furthermore it can also detect classes of neuroprotective agents that can show to be neuroprotective in longer-term classical EAE studies [30]. It has previously been found that housing conditions can influence the incidence of spontaneous EAE in some myelin-specific T cell receptor transgenic mice [14]. Therefore, differences in housing conditions may exhibit subtle influences on disease course. However, the disease incidence in the double transgenic reported here is consistent with that reported in disease induced with *Bordetella pertussis* toxin in single MOG-specific TCR transgenic mice [11, 17). This suggests that the protocol defined here will be robust and translatable to different laboratories.

Although models are developed to simulate aspects of the human disease, this new model may also offer significant advantages over existing EAE models (see Table 1). Most notably, there may be a significant refinement in accordance with the 3Rs principle of replacement, refinement and reduction of animals in research. Currently, the EAE induction protocol is considered to be a severe/substantial, United Kingdom Home Office procedure and so the introduction of the milder, MOG^TCR^x*Thy1*CFP mouse model allows disease processes to be studied in animals without the necessity of using Freund’s adjuvant that can cause discomfort and granulomas at the injection site or the development of weight loss and paralysis [8]. The current model uses a milder and quicker induction process that allows one to reduce the number of animals used in each experiment and the outcomes can be obtained in a shorter period of time than outcomes using conventional EAE. Animals are in the experiment for weeks versus weeks to months for classic EAE experiments [8], with both humane and economic benefits. The disability in this model is limited to visual sensory loss, which could occur as part of an EAE experiment. It is important to stress in this context that sight is a sense that is not of primary importance to rodents as they have evolved to be active in the dark and have poor visual sensitivity. Many laboratory strains of mice such as SJL and CBA mice have hereditary retinal dystrophy, which does not affect normal behaviour [31]. As the model has been developed on the C57BL/6 background, commonly used for the generation of transgenic mice and readily available from commercial breeders, this should help the rapid dissemination and adoption of this model by other groups. Many EAE studies only use paralysis as a read-out for the assessment of whether a transgene null mutation or a drug influences disease; this would be difficult to justify ethically with the availability of alternatives that are milder and more humane, such as the model developed here.

**Table 1:** Comparison of classical EAE with the novel optic neuritis model.^a^ Refinement and reduction opportunities, economic benefits and application of the novel optic neuritis model in comparison to classical EAE.

	**Classical EAE**	**Optic Neuritis Model**
*Induction Protocol (REFINEMENT)*	Uses Freund’s adjuvant	Avoids Freund’s adjuvant
*Disease severity (REFINEMENT)*	Paralytic disease	Non-paralytic disease
	Additional signs present	No additional signs present
	Long time in procedure	Less time in procedure
	SUBSTANTIAL/SEVERE procedure	MODERATE/MILD procedure
*Outcome measures (REFINEMENT AND REDUCTION)*	Subjective outcomes	Objective outcomes
	Non-parametric statistics	Parametric statistics (more power)
	Lethal outcome measures	Non-lethal outcome measures
	One-time point histological	Repeated serial assessment in vivo
	Outcome requires culling	Group size reductions
*Application*	Immunosuppressive agents	Immunosuppressive agents
	Neuroprotective agents	Neuroprotective agents
	Neurorepair agents (histology)	Neurorepair agents (electrophysiology)
*Speed*	Results in 3-4 weeks (immunosuppression)	Results in 2-3 weeks
	Results in 2-3 months (Neuroprotection)	
	Analysis in days/weeks	Analysis in minutes/hours
	Histological analysis in weeks	
*Consumable costs*	Animal costs for 2-3 months	Animal costs for 2-3 weeks
	High recurrent costs	Lower recurrent costs
*Human costs*	Weekdays, weekends and bank holidays checking animals	1-2 hours a day extra free, free weekends and bank holidays
*Solid science*	Non-parametric outcomes	Parametric outcomes
	Histological outcomes	Non-histological outcomes
	Observer influence	Observer- independent
		Human relevant outcomes

^a^Refinement and reduction opportunities, economic benefits and application of the novel optic neuritis model in comparison to classical EAE.

Furthermore there are many other merits that justify the use of this model over existing EAE models, in particular potential economic benefits. This can be achieved through reduced costs of labour and monitoring. Animals undergoing EAE require daily monitoring, particularly during periods of paralysis, to ensure humane endpoints are adequately monitored [8]. In addition there may be benefit in animal husbandry costs through a reduction in numbers of animals used or by a reduction in the time in procedure, which may be twice as long to monitor neuroprotective treatments [8]. One advantage of using EAE is that it has an easily visualised neurological problem that varies and can be monitored over time. However, the neurological outcomes are typically based on subjective, often observer-dependent, non-linear and non-parametric neurological scales that require a large number of observations to achieve statistical power, compared to objective parametric outcomes that are observer-independent. Furthermore analysis of EAE often uses single point-in-time analysis following terminal procedures/killing of animals and costly and time-consuming histology, which is again subjective and semi-quantitative focusing on a few select areas of the CNS. This new ON model allows non-lethal, serial and relatively quick monitoring of clinically-relevant, quantitative outcome measures that will enable fewer animals to be used. 

Whilst this model can be applied to the assessment of immune function and neurorepair strategies using drugs or other transgenic or knockout mice, we have shown that it can also be used to examine neuroprotective strategies, an area of unmet clinical need in MS. The mechanisms of neuronal damage in multiple sclerosis and EAE are varied and results from direct attack and the influence of the inflammatory penumbra that occurs during active inflammation. This appears to condition a neurodegenerative environment associated with glial-driven inflammation, neural metabolic failures, which no longer responds to peripheral immunosuppression both in MS and in progressive EAE [32,33]. In this current model damage is generated from the inflammatory penumbra and can model events in MS. Sodium channel blockade with OXC was found to be neuroprotective and prevented the RGC loss. The mechanism of neuroprotection is unlikely to be due to lymphoid cell immunosuppression based on our results of using sodium channel blockers in EAE [20]. Neuroprotection with OXC is most likely to occur at the level of the axon or nerve, or perhaps through an inhibition of microglial activity, which are probably central to the pathogenesis of progressive MS, as has been discussed extensively elsewhere [26,30] as these doses of drug are not T cell immunosuppressive such that they prevent disease developing [33]. However, in progressive disease additional benefit may be achieved through limitation of sodium loading in nerves, particularly demyelinated nerves that can lead to excitotoxicity and calcium induced apoptosis [32]. We aim to translate these animal studies into optic neuritis MS, where a similar inflammatory penumbra develops. Because of the ability to induce demyelination and to image the fate of individual nerve cells, future studies will be able to monitor whether a slow neurodegeneration of demyelinated nerves, resembling progressive MS develops. There we have found that sodium channel blockage appears to be neuroprotective in drug compliant people with secondary progressive MS, supporting the translatability from EAE [34,35]. Biomarker report from the phase II lamotrigine trial in secondary progressive MS-neurofilament as a surrogate of disease progression [35]. These results provide promising evidence to support sodium channel blockers as a neuroprotective agent in optic neuritis and MS.

Although the first large scale clinical trial of a sodium channel blocker, Lamotrigine, in MS failed to show any significant benefit [36]. A main reason for the failure of the trial was the inability of MRI as primary outcome measure to effectively detect axonal damage and neuronal loss, which was masked by drug-induced pseudoatrophy [37]. Another reason is that the wrong cohort of people with MS was studied. This trial enrolled people with advanced secondary progressive disease and who would have already lost much of their compensatory pool of axons, such that inhibition of salutatory conduction led to poor tolerance of the drug and non-adherence [36]. Slow progression of neurodegeneration that is independent of inhibition of autoimmunity can be modelled in EAE [9,38], but most EAE paradigms are more reflective of neurodegeneration driven by active inflammation. Whilst further studies are required to determine the long-term fate of demyelinated axons, it is possible to translate the current findings from this mouse model towards the treatment of human disease, such as acute ON in MS. Indeed the development work obtained during this project has helped inform the development of a novel trial design that we developed for the use of a sodium channel blocker in acute ON (NCT01451593).

ON is frequently associated with MS and often precedes other neurologic deficits associated with MS [1]. Although the majority of people with MS experience persistent visual defects following ON, gross visual acuity as measured with a Snellen chart typically returns to normal and therefore people may be more reluctant to undergo intra-ocular injection as proof of principle for the development of siRNA approaches. However, frequent intra-ocular injections are generally well tolerated by people with diseases such as macular degeneration. Neuromyelitis optica/Devic’s disease is an autoimmune disease involving the spinal cord and optic nerve that is associated with autoantibodies against aquaporin-4 [39] and interestingly antibodies against MOG [40]. ON attacks occur in neuromyelitis optica, but they result in significantly worse visual outcome than MS and the majority of people will show some visual loss and risk the development of blindness [39,41]. As such this novel siRNA-based approach could be developed towards the treatment of neuromyelitis optica, where people may be more willing to have invasive treatments directed towards the eye. This use of siRNA holds potential as a novel therapeutic approach to treating ON and demonstrates that it is possible to induce a functional knock-down in the visual pathway as was found previously following optic nerve crush [20]. This route of delivery provides a powerful approach to neurobiology such that it may be possible to explore the local knock-down of proteins that would otherwise be lethal in global knockout mice. Currently there are a number of siRNA-based drugs in the pipeline for human use, including the siRNA to caspase 2 (QPI-1007) used in this study. Whilst optic neuritis in multiple sclerosis may not be the major first indication because in general sight returns, however this could be examined in Devics MS or neuromyelitis optica associated with the development of aquaporin-4 and also MOG-specific antibodies where blindness is the usual outcome. Therefore a sight saving treatment may be considered and is currently being explored.

In summary, the generation and use of the MOG^TCR^x*Thy1*CFP model has many implications for the work undertaken in this field of experimental neuroscience research and the development of drugs for people with MS. This new model provides a more humane and rapid screening tool for the translation of treatments for neuroimmunological diseases and has demonstrated the potential of two neuroprotective therapies.

## Supporting Information

Table S1 **Development of neurological EAE in MOGTCR mice induced to develop ON following administration of different concentrations of MOG-specific mAb.** MOGTCR mice were immunised with PTX (Day 0 and 2) followed by 0.1mg (n=8), 0.5mg (n=7) or 1.0mg (n=5) Z12 MOG-specific mAb (Day 14) and were sacrificed on Day 21 post-disease induction. Neurological EAE score was assessed daily and % incidence of neurological EAE was calculated by the number of animals developing any signs of neurological EAE (score 1-5) compared to the total number of animals in the group. The results show the maximal EAE clinical score of all animals within the group and the RGC density.
(DOCX)

## References

[1] Compston A, Coles A (2008) Multiple sclerosis. Lancet 372: 1502–1517. doi:10.1016/S0140-6736(08)61620-7. PubMed: 18970977. 18970977

[2] Bjartmar C, Wujek JR, Trapp BD (2003) Axonal loss in the pathology of MS: consequences for understanding the progressive phase of the disease. J Neurol Sci 206: 165–171. doi:10.1016/S0022-510X(02)00069-2. PubMed: 12559505. 12559505

[3] Pryce G, O’Neill JK, Croxford JL, Amor S, Hankey DJ et al. (2005) Autoimmune tolerance eliminates relapses but fails to halt progression in a model of multiple sclerosis. J Neuroimmunol 165: 41–52. doi:10.1016/j.jneuroim.2005.04.009. PubMed: 15939483. 15939483

[4] Cohen JA, Reingold SC, Polman CH, Wolinsky JS (2012) Disability outcome measures in multiple sclerosis clinical trials: current status and future prospects. Lancet Neurol 11: 467–476. doi:10.1016/S1474-4422(12)70059-5. PubMed: 22516081. 22516081

[5] Talman LS, Bisker ER, Sackel DJ, Long DA Jr., Galetta KM et al. (2010) Longitudinal study of vision and retinal nerve fiber layer thickness in multiple sclerosis. Ann Neurol 67: 749–760. PubMed: 20517936. 20517936 10.1002/ana.22005 PMC2901775

[6] Baker D, Gerritsen W, Rundle J, Amor S (2011) Critical appraisal of animal models of multiple sclerosis. Mult Scler 17: 647–657. doi:10.1177/1352458511398885. PubMed: 21372117. 21372117

[7] Steinman L, Zamvil SS (2006) How to successfully apply animal studies in experimental allergic encephalomyelitis to research on multiple sclerosis. Ann Neurol 60: 12–21. doi:10.1002/ana.20913. PubMed: 16802293. 16802293

[8] Al-Izki S, Pryce G, Jackson SJ (2012) Practical guide to the induction of relapsing progressive experimental autoimmune encephaolomyelitis in the Biozzi ABH mouse. Multiple Sclerosis Relat Disord 1: 29–38. doi:10.1016/j.msard.2011.09.001. 25876448

[9] Hampton DW, Anderson J, Pryce G, Irvine K-A, Giovannoni G et al. (2008) An experimental model of secondary progressive multiple sclerosis that shows regional variation in gliosis, remyelination, axonal and neuronal loss. J Neuroimmunol 201-202: 200–211. doi:10.1016/j.jneuroim.2008.05.034. PubMed: 18672298. 18672298

[10] O’Neill JK, Baker D, Morris MM, Gschmeissner SE, Jenkins HG et al. (1998) Optic neuritis in chronic relapsing experimental allergic encephalomyelitis in Biozzi ABH mice: Demyelination and fast axonal transport changes in disease. J Neuroimmunol 82: 210–218. doi:10.1016/S0165-5728(97)00203-8. PubMed: 9585818. 9585818

[11] Guan Y, Shindler KS, Tabuena P, Rostami AM (2006) Retinal ganglion cell damage induced by spontaneous autoimmune optic neuritis in MOG-specific TCR transgenic mice. J Neuroimmunol 178: 40–48. doi:10.1016/j.jneuroim.2006.05.019. PubMed: 16828169. 16828169

[12] Bettelli E, Baeten D, Jäger A, Sobel RA, Kuchroo VK (2006) Myelin oligodendrocyte glycoprotein-specific T and B cells cooperate to induce a Devic-like disease in mice. J Clin Invest 116: 2393–2402. doi:10.1172/jci28334. PubMed: 16955141. 16955141 PMC1555670

[13] Feng G, Mellor RH, Bernstein M, Keller-Peck C, Nguyen QT et al. (2000) Imaging Neuronal Subsets in Transgenic Mice Expressing Multiple Spectral Variants of GFP. Neuron 28: 41–51. doi:10.1016/S0896-6273(00)00084-2. PubMed: 11086982. 11086982

[14] Goverman J, Woods A, Larson L, Weiner LP, Hood L, Zaller DM (1993) Transgenic mice that express a myelin basic protein-specific T cell receptor develop spontaneous autoimmunity.Cell 72(4): 551-560. doi:10.1016/0092-8674(93)90074-Z. PubMed: 7679952. 7679952

[15] Svingen T, Spiller CM, Kashimada K, Harley VR, Koopman P (2009) Identification of Suitable Normalizing Genes for Quantitative Real-Time RT-PCR Analysis of Gene Expression in Fetal Mouse Gonads. Sexual Dev 3: 194–204. doi:10.1159/000228720. PubMed: 19752599. 19752599

[16] Piddlesden SJ, Lassmann H, Zimprich F, Morgan BP, Linington C (1993) The demyelinating potential of antibodies to myelin oligodendrocyte glycoprotein is related to their ability to fix complement. Am J Pathol 143: 555–564. PubMed: 7688186. 7688186 PMC1887024

[17] Bettelli E, Pagany M, Weiner HL, Linington C, Sobel RA et al. (2003) Myelin oligodendrocyte glycoprotein-specific T cell receptor transgenic mice develop spontaneous autoimmune optic neuritis. J Exp Med 197: 1073–1081. doi:10.1084/jem.20021603. PubMed: 12732654. 12732654 PMC2193967

[18] Thomas BB, Seiler MJ, Sadda SR, Coffey PJ, Aramant RB (2004) Optokinetic test to evaluate visual acuity of each eye independently. J Neurosci Methods 138: 7–13. doi:10.1016/j.jneumeth.2004.03.007. PubMed: 15325106. 15325106

[19] Leung CKS, Lindsey JD, Chen L, Liu Q, Weinreb RN (2009) Longitudinal profile of retinal ganglion cell damage assessed with blue-light confocal scanning laser ophthalmoscopy after ischaemic reperfusion injury. Br J Ophthalmol 93: 964–968. doi:10.1136/bjo.2008.150482. PubMed: 19224902. 19224902 PMC5499383

[20] Wang X, Archibald ML, Stevens K, Baldridge WH, Chauhan BC (2010) Cyan fluorescent protein (CFP) expressing cells in the retina of Thy1-CFP transgenic mice before and after optic nerve injury. Neurosci Lett 468: 110–114. doi:10.1016/j.neulet.2009.10.077. PubMed: 19879331. 19879331

[21] Ahmed Z, Doward AI, Pryce G, Taylor DL, Pocock JM et al. (2002) A Role for Caspase-1 and -3 in the Pathology of Experimental Allergic Encephalomyelitis : Inflammation Versus Degeneration. Am J Pathol 161: 1577–1586. doi:10.1016/S0002-9440(10)64436-7. PubMed: 12414506. 12414506 PMC1850770

[22] Morris-Downes MM, Smith PA, Rundle JL, Piddlesden SJ, Baker D et al. (2002) Pathological and regulatory effects of anti-myelin antibodies in experimental allergic encephalomyelitis in mice. J Neuroimmunol 125: 114–124. doi:10.1016/S0165-5728(02)00040-1. PubMed: 11960647. 11960647

[23] Talman LS, Bisker ER, Sackel DJ, Long DA, Galetta KM et al. (2010) Longitudinal study of vision and retinal nerve fiber layer thickness in multiple sclerosis. Ann Neurol 67: 749–760. doi:10.1002/ana.22005. PubMed: 20517936. 20517936 PMC2901775

[24] Galetta KM, Calabresi PA, Frohman EM, Balcer LJ (2011) Optical coherence tomography (OCT): imaging the visual pathway as a model for neurodegeneration. Neurother J American Society For Experimental NeuroTherapeutics 8: 117–132. doi:10.1007/s13311-010-0005-1. PubMed: 21274691. PMC3075740 21274691

[25] Coles AJ, Wing MG, Molyneux P, Paolillo A, Davie CM et al. (1999) Monoclonal antibody treatment exposes three mechanisms underlying the clinical course of multiple sclerosis. Ann Neurol 46: 296–304. doi:10.1002/1531-8249(199909)46:3. PubMed: 10482259. 10482259

[26] Waxman SG (2008) Mechanisms of Disease: sodium channels and neuroprotection in multiple sclerosis[mdash]current status. Nat Clin Pract Neurol 4: 159–169. doi:10.1038/ncpneuro0735. PubMed: 18227822. 18227822

[27] Raju K, Meirion D, Paul AB, Susan MH, Kenneth JS (2003) Blockers of sodium and calcium entry protect axons from nitric oxide-mediated degeneration. Ann Neurol 53: 174–180. doi:10.1002/ana.10443. PubMed: 12557283. 12557283

[28] Kleinewietfeld M, Manzel A, Titze J, Kvakan H, Yosef N et al. (2013) Sodium chloride drives autoimmune disease by the induction of pathogenic TH17 cells. Nature 496(7446): 518-522. doi:10.1038/nature11868. PubMed: 23467095. 23467095 PMC3746493

[29] Coquet JM, Middendorp S, van der Horst G, Kind J, Veraar EA, Xiao Y et al. (2013) The CD27 and CD70 costimulatory pathway inhibits effector function of T helper 17 cells and attenuates associated autoimmunity.Immunity 38: 53-65. doi:10.1016/j.immuni.2012.09.009. PubMed: 23159439. 23159439

[30] Black JA, Waxman SG (2012) Sodium channels and microglial function. Exp Neurol 234: 302–315. doi:10.1016/j.expneurol.2011.09.030. PubMed: 21985863. 21985863

[31] Hafezi F, Grimm C, Simmen BC, Wenzel A, Remé CE (2000) Molecular ophthalmology: an update on animal models for retinal degenerations and dystrophies. Br J Ophthalmol 84: 922–927. doi:10.1136/bjo.84.8.922. PubMed: 10906106. 10906106 PMC1723576

[32] Dutta R, Trapp BD (2011) Mechanisms of Neuronal Dysfunction and Degeneration in Multiple Sclerosis. Prog Neurobiol 93: 1-12. doi:10.1016/j.pneurobio.2010.09.005. PubMed: 20946934. 20946934 PMC3030928

[33] Al-Izki S, Pryce G, Amor S, Gerritsen W, Garthwaite J et al. (2011) Selective targeting of neuroprotection to MS lesions: sodium channel blockers in experimental autoimmune encephalomyelitis. Mult Scler 17: S209.

[34] Bechtold DA, Miller SJ, Dawson AC, Sun Y, Kapoor R et al. (2006) Axonal protection achieved in a model of multiple sclerosis using lamotrigine. J Neurol 253(12): 1542-1551. doi:10.1007/s00415-006-0204-1. PubMed: 17219031. 17219031

[35] Gnanapavan S, Grant D, Morant S, Furby J, Hayton T et al. (2013) Biomarker report from the phase II lamotrigine trial in secondary progressive MS-neurofilament as a surrogate of disease progression. PLOS ONE 8(8): e70019. doi:10.1371/journal.pone.0070019. PubMed: 23936370. 23936370 PMC3731296

[36] Kapoor R, Furby J, Hayton T, Smith KJ, Altmann DR et al. (2010) Lamotrigine for neuroprotection in secondary progressive multiple sclerosis: a randomised, double-blind, placebo-controlled, parallel-group trial. Lancet Neurol 9: 681–688. doi:10.1016/S1474-4422(10)70131-9. PubMed: 20621711. 20621711

[37] Hayton T, Furby J, Smith K, Altmann D, Brenner R et al. (2011) Longitudinal changes in magnetisation transfer ratio in secondary progressive multiple sclerosis: data from a randomised placebo controlled trial of lamotrigine. J Neurol, 258: 1–10. PubMed: 21181182. 21904901 10.1007/s00415-011-6212-9

[38] Al-Izki S, Pryce G, Jackson SJ, Giovannoni G, Baker D (2011) Immunosuppression with FTY720 is insufficient to prevent secondary progressive neurodegeneration in experimental autoimmune encephalomyelitis. Multiple sclerosis (Houndmills, Basingstoke, England) 17: 939–948. doi:10.1177/1352458511400476. 21459808

[39] Oh J, Levy M (2012) Neuromyelitis optica: an antibody-mediated disorder of the central nervous system. Neurology Res International, 2012: 2012: 460825 doi:10.1155/2012/460825. PubMed: 22363840. PMC3272864 22363840

[40] Mader S, Gredler V, Schanda K, Rostasy K, Dujmovic I et al. (2011) Complement activating antibodies to myelin oligodendrocyte glycoprotein in neuromyelitis optica and related disorders. J Neuroinflammation 8: 184. doi:10.1186/1742-2094-8-184. PubMed: 22204662. 22204662 PMC3278385

[41] Fernandes DB, Ramos R, de IP, Falcochio C, Apóstolos-Pereira S, Callegaro D et al. (2012) Comparison of visual acuity and automated perimetry findings in patients with neuromyelitis optica or multiple sclerosis after single or multiple attacks of optic neuritis. J Neuro Ophthalmol Off J North American Neuro Ophthalmology Society 32: 102–106. doi:10.1097/WNO.0b013e31823a9ebc. 22157535

