# Supplementary material for: Neuroprotection in a Novel Mouse Model of Multiple Sclerosis
Source: PLoS One. 2013 Nov 4;8(11):e79188. doi: 10.1371/journal.pone.0079188 (PMC3817036; doi:10.1371/journal.pone.0079188)
Supplement: Table S1 — Development of neurological EAE in MOGTCR mice induced to develop ON following administration of different concentrations of MOG-specific mAb. MOGTCR mice were immunised with PTX (Day 0 and 2) followed by 0.1mg (n=8), 0.5mg (n=7) or 1.0mg (n=5) Z12 MOG-specific mAb (Day 14) and were sacrificed on Day 21 post-disease induction. Neurological EAE score was assessed daily and % incidence of neurological EAE was calculated by the number of animals developing any signs of neurological EAE (score 1-5) compared to the total number of animals in the group. The results show the maximal EAE clinical score of all animals within the group and the RGC density. (DOCX) [file pone.0079188.s001.docx]

| **Treatment** | **% MOG^TCR^x*Thy1*CFP mice developing clinical EAE** | **EAE Score (Mean ±SEM)** |
| --- | --- | --- |
| 0.0mg Z12 MOG-specific mAb | 0 | 0 |
| 0.1mg Z12 MOG-specific mAb | 25 | 0.9±0.6 |
| 0.5mg Z12 MOG-specific mAb | 38 | 1.3±0.7 |
| 1.0mg Z12 MOG-specific mAb | 60 | 3.0±0.7 |
